# WHF Roadmap on Secondary Prevention of CVD

**DOI:** 10.5334/gh.1294

**Published:** 2024-01-22

**Authors:** David A. Wood

**Affiliations:** 1 National Institute for Prevention and Cardiovascular Health, University of Galway, Republic of Ireland

**Keywords:** editorial, secondary prevention, WHF, roadmap

The concept of the WHF Roadmap was created by Professor Srinath Reddy, a former president of WHF, and through his leadership the WHF prioritised secondary prevention of CVD, hypertension detection, and treatment and tobacco control as the top three global targets with the biggest potential to reduce the burden of CVD. Patients who have already survived a heart attack or stroke are recommended as the highest priority for prevention because their absolute five-year risk of a further cardiovascular event at a population level is about 20% higher than patients without CVD and these patients with manifest disease account for up to half of all subsequent CVD events in the population [1].

The first Roadmap was launched on February 13^th^, 2015, at a Policy Forum on Secondary Prevention of CVD and published in Global Heart and followed by Roadmaps on Hypertension and Tobacco Control [2,3,4].

The raison d’être of these WHF Roadmaps is to translate science into health policy, define pathways to effective prevention, detection and treatment of different aspects of CVD, identify potential roadblocks along the way, and propose evidence-based solutions to overcome them. The overall aim of these Roadmaps is to stimulate the development of national policies to achieve the UN Sustainable Development Goal of a 30% reduction in premature mortality from non-communicable diseases, of which CVD is the major cause of premature death, disability, and health care expenditure globally, by 2030 [5]. This target can only be achieved if there is a substantial reduction in premature mortality from atherosclerotic cardiovascular disease, principally heart attacks and strokes, and especially in middle- and low-income countries, which bear the largest burden of CVD.

The goal of this updated Roadmap on Secondary Prevention of CVD is to drive national policies for prevention of CVD by identifying key barriers at an individual level, health care level and health policy level and to offer evidence-based solutions through a conceptual framework and health system approaches. This update draws on a wealth of new evidence from observational studies and randomised controlled trials summarised in the latest international CVD prevention guidelines and a consultation with WHF members.

At an individual level, the key roadblocks are lifestyle behaviour change, adherence to cardiovascular medications, and participation in cardiovascular prevention and rehabilitation programmes. At the health care level, inequalities to cardiovascular care, medicines, and vaccines are identified together with clinical inertia, defined as barriers in knowledge, attitudes, and behaviour of health care providers, and limited availability of local guidelines on CVD prevention. At a policy level, lack of investment in primary care is considered the first priority together with built-up urban environments, which are not supportive of physical activity, fast food outlets, which discourage healthy eating, and air pollution. Structural biases which are embedded within systems of societies, such as institutions, policies, and practices which stem from historical, cultural, and societal norms are also highlighted in relation to race and gender.

The key interventions and strategies recommended at the individual, health care systems, and policy level are described to overcome these roadblocks and, given this evidence base, it is expected these approaches will lead to better implementation of guideline recommendations for secondary prevention of CVD with improved patient outcomes.

At the individual level, improving health literacy is prioritised so that patients can access and process health information and make informed decisions about their own health and health care. Supporting self-management is also advocated in relation to making healthy lifestyle changes and adhering with cardioprotective medications.

For health care systems strategies are proposed to improve secondary prevention in hospital and primary care. Optimising the hospital discharge process after an acute CVD event is the first step with protocols and decision support systems in place that facilitate the prescription of secondary prevention medicines, lifestyle recommendations, and referral to cardiovascular prevention and rehabilitation programmes. Following discharge, facilitating access to primary health care services is considered key to improving health outcomes in secondary prevention over the longer term. At the health care level it is also necessary to strengthen the delivery of secondary prevention services based on guidelines, training of health professionals with the knowledge, skills, and attitudes to implement these guidelines and given the resources and time to deliver preventive care. Multidisciplinary expertise is required and health workers, other than professionally qualified health care staff, need to be trained with these essential skills given the global shortage of health care workers. Alternate models of secondary prevention to traditional hospital based services need to be explored including community, home-based, and digital technologies. Finally the outcomes of these different evidence-based approaches to secondary prevention need to be audited so that gaps in care can be addressed.

With the arrival of the Covid-19 pandemic, the development of digital cardiac rehabilitation really accelerated given that many programmes around the world were no longer able to see patients face-to-face. This has involved phone calls, text messaging, smartphone apps, virtual reality, and wearable and wireless monitoring devices to deliver patient education, behavioural change support, including remote exercise supervision, risk factor management, and psychosocial support. Short-term outcomes in terms of lifestyle and risk factor improvements are encouraging, but trials demonstrating hard outcomes with cost effectiveness are required in different health care settings.

At a health policy level, a national action plan for secondary CVD prevention is required. In this context fiscal policies like health taxes levied on tobacco products, alcoholic, and sugar-sweetened beverages can result in healthier lives and generate revenue. As part of the national action plan public health policies are critical to reducing population smoking rates, promoting healthy eating, encouraging physical activity, and improving air quality. Cardioprotective medications in the form of good quality, safe, and inexpensive generics are central to secondary prevention and equitable access around the world is required for all patients. Fixed-dose combination therapies, by combining multiple active pharmaceutical ingredients, improve medication adherence as well as risk factor control. The polypill strategy – aspirin, ramipril and atorvastatin – in secondary prevention has also reduced major adverse cardiovascular events and needs to be included in the WHO Model List of Essential Medicines alongside other combination therapies.

The WHF Roadmaps, of which the Secondary Prevention Roadmap in 2015 was the prototype, provide a unique approach for policy makers and health professionals to define their own national pathways to care, the roadblocks to delivering this evidence based care and solutions, all of which need to be tailored to different national contexts. The first and most important step is political as all governments need to create their own national policy for CVD prevention which includes secondary, primary, and primordial prevention. The specific role of National Societies of Cardiology working with other specialist societies, such as stroke, hypertension, lipids, diabetes, and kidney disease, is to develop or adapt evidence based guidelines on CVD prevention to inform this national policy with clearly defined priorities and patient lifestyle, risk factor and therapeutic targets. The top priority in this context should be secondary prevention of atherosclerotic CVD – coronary heart disease, cerebrovascular disease, aortic and peripheral arterial disease – as these patients usually present with symptomatic disease, are treated by hospital services, and are at greatest risk of a further cardiovascular event. The modes and settings of secondary preventive care – individual, group or virtual in hospital, community, or home – need to be defined together with the health care professionals and other workers who are going to deliver care as a team. Training of these health care professionals is required and their care needs to be quality assured. The chosen modes of care and the teams who are going to deliver it need to be nationally resourced. Auditing national patient outcomes for lifestyle, attainment of risk factor targets, and adherence with medications is essential to identifying gaps in implementation in relation to guideline targets and how these can be closed.

Every country faces, to a greater or lesser extent, the intolerable social and economic burden of premature mortality from CVD and, even though the evidence for secondary prevention is much the same worldwide, each country Roadmap will necessarily be different given societal differences, although based on the same principals. I suggest, as President of a National Society of Cardiology or National Heart Foundation, that your first question to your government is: ‘Do we have a national policy for prevention of cardiovascular disease?’ If the answer is ‘No’ then you, your board and your society or foundation need to lobby government for one, and this WHF Roadmap on Secondary Prevention is your starter blueprint.
